# Coronary Hemodynamics in Patients With Severe Aortic Stenosis and Coronary Artery Disease Undergoing Transcatheter Aortic Valve Replacement: Implications for Clinical Indices of Coronary Stenosis Severity

**DOI:** 10.1016/j.jcin.2018.07.019

**Published:** 2018-10-22

**Authors:** Yousif Ahmad, Matthias Götberg, Christopher Cook, James P. Howard, Iqbal Malik, Ghada Mikhail, Angela Frame, Ricardo Petraco, Christopher Rajkumar, Ozan Demir, Juan F. Iglesias, Ravinay Bhindi, Sasha Koul, Nearchos Hadjiloizou, Robert Gerber, Punit Ramrakha, Neil Ruparelia, Nilesh Sutaria, Gajen Kanaganayagam, Ben Ariff, Michael Fertleman, Jon Anderson, Andrew Chukwuemeka, Darrel Francis, Jamil Mayet, Patrick Serruys, Justin Davies, Sayan Sen

**Affiliations:** a National Heart and Lung Institute, Hammersmith Hospital, Imperial College London, London, United Kingdom; b Department of Cardiology, Clinical Sciences, Lund University, Skåne University Hospital, Sweden; c Department of Cardiology, Hammersmith Hospital, Imperial College Healthcare NHS Trust, London, United Kingdom; d Cardiology Department, Lausanne University Hospital, Lausanne, Switzerland; e Department of Cardiology, Royal North Shore Hospital, Sydney, Australia; f Department of Cardiology, Conquest Hospital, St. Leonards-on-Sea, United Kingdom

**Keywords:** aortic stenosis, coronary flow, fractional flow reserve, instantaneous wave-free ratio, TAVR, AS, aortic stenosis, CAD, coronary artery disease, FFR, fractional flow reserve, FFR-flow, whole-cycle hyperemic flow, iFR, instantaneous wave-free ratio, iFR-flow, flow during the wave-free period of diastole, LV, left ventricular, MVR, microvascular resistance, PdPa-flow, whole-cycle resting flow, TAVR, transcatheter aortic valve replacement

## Abstract

**Objectives:**

In this study, a systematic analysis was conducted of phasic intracoronary pressure and flow velocity in patients with severe aortic stenosis (AS) and coronary artery disease, undergoing transcatheter aortic valve replacement (TAVR), to determine how AS affects: 1) phasic coronary flow; 2) hyperemic coronary flow; and 3) the most common clinically used indices of coronary stenosis severity, instantaneous wave-free ratio and fractional flow reserve.

**Background:**

A significant proportion of patients with severe aortic stenosis (AS) have concomitant coronary artery disease. The effect of the valve on coronary pressure, flow, and the established invasive clinical indices of stenosis severity have not been studied.

**Methods:**

Twenty-eight patients (30 lesions, 50.0% men, mean age 82.1 ± 6.5 years) with severe AS and coronary artery disease were included. Intracoronary pressure and flow assessments were performed at rest and during hyperemia immediately before and after TAVR.

**Results:**

Flow during the wave-free period of diastole did not change post-TAVR (29.78 ± 14.9 cm/s vs. 30.81 ± 19.6 cm/s; p = 0.64). Whole-cycle hyperemic flow increased significantly post-TAVR (33.44 ± 13.4 cm/s pre-TAVR vs. 40.33 ± 17.4 cm/s post-TAVR; p = 0.006); this was secondary to significant increases in systolic hyperemic flow post-TAVR (27.67 ± 12.1 cm/s pre-TAVR vs. 34.15 ± 17.5 cm/s post-TAVR; p = 0.02). Instantaneous wave-free ratio values did not change post-TAVR (0.88 ± 0.09 pre-TAVR vs. 0.88 ± 0.09 post-TAVR; p = 0.73), whereas fractional flow reserve decreased significantly post-TAVR (0.87 ± 0.08 pre-TAVR vs. 0.85 ± 0.09 post-TAVR; p = 0.001).

**Conclusions:**

Systolic and hyperemic coronary flow increased significantly post-TAVR; consequently, hyperemic indices that include systole underestimated coronary stenosis severity in patients with severe AS. Flow during the wave-free period of diastole did not change post-TAVR, suggesting that indices calculated during this period are not vulnerable to the confounding effect of the stenotic aortic valve.

## Graphical abstract

**Figure:**
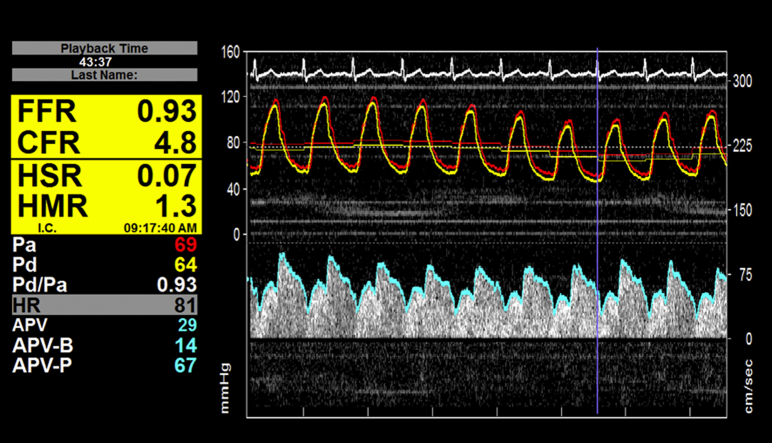

A significant proportion of patients with severe aortic stenosis (AS) have concomitant coronary artery disease (CAD) 1, 2. Determining the significance of CAD is challenging because traditional noninvasive and invasive indices of ischemia have not been validated in this setting (3). At present the decision to revascularize a coronary lesion in a patient with severe AS is based on angiography (3). This anatomic approach is unlikely to correctly identify those lesions that are truly flow limiting and may therefore lead to inappropriate treatment decisions (4).

Invasive indices of coronary stenosis severity provide more accurate localization of ischemia than noninvasive indices (5). There are several invasive indices of coronary artery stenosis severity. These are measured either during resting or hyperemic conditions and can be further divided into those that use the complete cardiac cycle (Pd/Pa, fractional flow reserve [FFR] [5]) or only a period within diastole (instantaneous wave-free ratio [iFR] [6]).

To validate whether an invasive index is accurate in determining lesion significance, in patients with severe AS, an understanding of how AS affects coronary flow is required. Transcatheter aortic valve replacement (TAVR) permits unique insights into the acute effects of AS on coronary physiology (7) because intracoronary physiology assessment can be made immediately before and after valve insertion, thereby minimizing any potential confounding factors. In this study, we aimed to use the TAVR model to determine how AS affects 1) phasic coronary flow; 2) hyperemic coronary flow; and 3) the most common clinically used indices of coronary stenosis severity, iFR and FFR.

## Methods

### Patient population

Twenty-eight consecutive patients (30 lesions) with severe AS planned for TAVR and moderate to severe CAD were included. Recruiting centers were the Hammersmith Hospital, Imperial College NHS Trust (London, United Kingdom) and Skane University Hospital (Lund, Sweden). TAVR was indicated by international guidelines (3), and the treatment decision was made at a heart team meeting. Exclusion criteria were known nonviable myocardium in the area of the corresponding coronary artery being studied, contraindication to the administration of adenosine, inability to consent, and weight more than 200 kg. All participants gave written informed consent, and the study was given full ethical approval (14/SC/1103).

### Cardiac catheterization protocol

**Figure 1:**
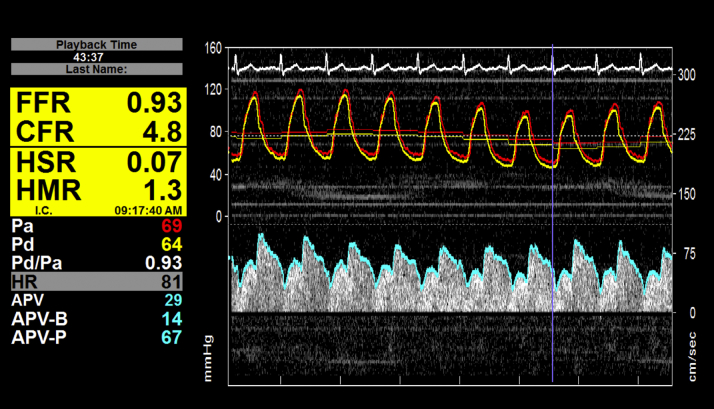
Figure Demonstrating an Example of Invasive Pressure and Doppler Flow Measurements APV = average peak flow velocity; APV-B = average peak flow velocity at baseline; APV-P = average peak flow velocity at peak hyperemia. CFR = coronary flow reserve; FFR = fractional flow reserve; HMR = hyperemic microvascular resistance; HR = heart rate; HSR = hyperemic stenosis resistance; Pa = aortic pressure; Pd = distal coronary pressure.

Cardiac catheterization and coronary angiography were undertaken via either the transradial or transfemoral route at the operator’s discretion, using standard equipment. A guiding catheter was used to intubate the vessel of interest. Heparin (5,000 U) was administered intra-arterially. A dual-pressure and Doppler sensor–equipped 0.014-inch guidewire was used for all physiological assessments (ComboWire, Volcano, San Diego, California). The guidewire signal was normalized in the aorta and then advanced a minimum of 3 vessel diameters distal to the stenosed segment. After an optimal and stable flow velocity signal was obtained, resting pressure and flow measurements were recorded. Hyperemia was then induced using a 150-μg bolus of intracoronary adenosine, and hyperemic measurements were made. At the end of each recording, the pressure sensor was returned to the catheter tip to ensure that there was no pressure drift. When drift was identified (≥0.01), all measurements were repeated. Left ventricular end-diastolic pressure (LVEDP) was recorded using a pigtail catheter placed in the LV cavity. The entire protocol was repeated immediately following the deployment of the new aortic valve. An example of an invasive pressure and flow trace is shown in Figure 1.

### TAVR procedure

All TAVR procedures were undertaken according to standard clinical protocols. All patients were treated under local anesthesia. The valves used were the balloon-expandable Edwards SAPIEN XT or S3 valve (Edwards Lifesciences, Irvine, California), the self-expandable Medtronic CoreValve or Evolut R valve (Medtronic, Minneapolis, Minnesota), or the repositionable Lotus valve (Boston Scientific, Natick, Massachusetts); valve choice was at the operator’s discretion.

### Analysis of hemodynamic data

The hemodynamic signals were processed using the associated instrument console (ComboMap, Volcano) and stored for offline analysis. Analog output feeds were taken from the pressure-velocity console and electrocardiograph, fed into a DAQ-Card AI-16E-4 (National Instruments, Austin, Texas), and acquired at 1 kHz with LabVIEW (National Instruments). Data were analyzed offline using a custom software package designed with MATLAB (The MathWorks, Natick, Massachusetts).

Coronary flow velocity (centimeters per second) was measured at baseline and during hyperemia.

Definitions of hemodynamic variables were as follows:

FFR = Pd_h_/Pa_h_ (5)
iFR = Pd_wfp_/Pa_wfp_ (6)
Flow during the wave-free period of diastole (iFR-flow) = *v*_wfp_
Whole-cycle hyperemic flow (FFR-flow) = *v*_h_
Whole-cycle resting flow (PdPa-flow) = *v*_b_
Systolic flow = *v*_systole_
Hyperemic microvascular resistance (MVR) = Pd_h_/*v*_h_ (8)
Basal MVR = Pd_b_/*v*_b_ (8)
iFR resistance = Pd_wfp_/*v*_wfp_ (9)
Systolic resistance = Pd_systole_/*v*_systole_
Basal stenosis resistance = ΔP_b_/*v*_b_ (10)
Hyperemic stenosis resistance = ΔP_h_/*v*_h_ (11)

where Pa is mean aortic pressure; Pd is mean intracoronary pressure distal to a stenosis; wfp is the wave-free period of diastole; *v*_h_ is mean flow velocity distal to a stenosis during hyperemia; *v*_b_ is mean flow velocity distal to a stenosis at baseline; ΔP_h_ is Pa − Pd during hyperemia; and ΔP_b_ is Pa − Pd at baseline.

**Figure 2:**
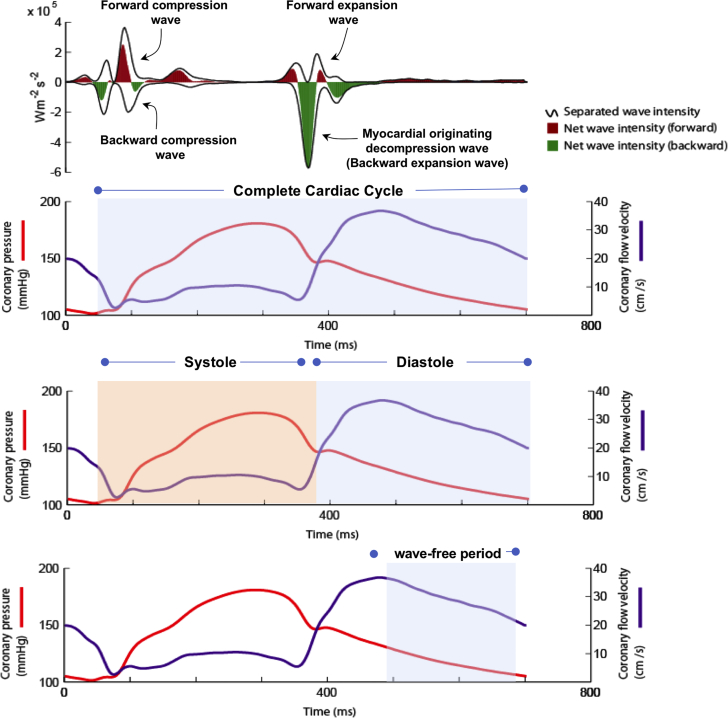
Outline of the Definitions and Calculations Used for Hemodynamic Parameters Used in the Phasic Analysis

Phasic analysis was performed to identify pressure and flow characteristics during different periods of the cardiac cycle. The wave-free period was identified using wave-intensity analysis as previously described (12). A custom-written MATLAB algorithm was used to separate systole, diastole, and the wave-free period to facilitate phasic analysis of hemodynamic data. A schematic outlining how this was performed is shown in Figure 2.

### Statistical analysis

Continuous variables are presented as mean ± SD unless otherwise stated. Comparisons before and after TAVR were performed using a Wilcoxon signed rank test. The threshold for statistical significance was set at 0.05.

## Results

### Patient population and procedural characteristics

Twenty-eight patients (30 lesions, 50.0% men, mean age 82.1 ± 6.5 years) were included. Baseline clinical characteristics are summarized in Table 1. Data regarding quantitative coronary angiography are shown in Table 2. The baseline echocardiographic and procedural characteristics are summarized in Table 3. Mean peak aortic valve velocity was 407.18 ± 93.68 cm/s, and mean peak gradient was 70.01 ± 32/85 mm Hg with a calculated mean aortic valve area of 0.68 ± 0.22 cm^2^ (velocity-time integral method).

**Table 1:** Baseline Clinical Characteristics

Age (yrs)	82.1 ± 6.5
Male	14 (50.0)
Body mass index (kg/m^2^)	27.90 ± 4.8
Diabetes	7 (25.0)
Hypertension	16 (57.1)
Hyperlipidemia	19 (67.9)
Former smokers	10 (35.7)
Current smokers	0 (0)
Previous myocardial infarction	1 (3.6)
Previous percutaneous coronary intervention	5 (17.9)
Previous coronary artery bypass grafting	1 (3.6)

Values are mean ± SD or n (%).

**Table 2:** Quantitative Coronary Angiographic Data

Target vessel (LAD/LCx/RCA)	16/7/7
Stenosis location (proximal/mid/distal)	12/18/0
Diameter stenosis by QCA (%)	56.11 ± 12.2
Area stenosis by QCA (%)	79.15 ± 10.7
Stenosis length (mm)	18.54 ± 5.4
Minimum luminal diameter (mm)	1.16 ± 0.4
Minimum luminal area (mm^2^)	1.20 ± 0.9

Values are n or mean ± SD.

LAD = left anterior descending coronary artery; LCx = left circumflex coronary artery; QCA = quantitative coronary angiography; RCA = right coronary artery.

**Table 3:** Baseline Echocardiographic and Procedural Characteristics

	Pre-TAVR	Post-TAVR	p Value
Peak velocity (cm/s)	407.18 ± 93.68	209.58 ± 46.0	<0.001
Peak gradient (mm Hg)	70.01 ± 32.85	17.58 ± 7.3	<0.001
Mean gradient (mm Hg)	37.64 ± 18.48	8.93 ± 4.2	<0.001
Aortic valve area (cm^2^)	0.68 ± 0.22	1.48 ± 0.4	<0.001
LV systolic function			
Normal	20 (71.4)	20 (71.4)	NS
Mildly impaired	3 (10.7)	3 (10.7)	NS
Moderately impaired	2 (7.1)	4 (14.3)	NS
Severely impaired	3 (10.7)	1 (3.6)	NS
LV end-diastolic pressure (mm Hg)	17.63 ± 7.9	15.44 ± 6.6	0.06
Paravalvular leak			
None		15 (53.6)	
Mild		13 (46.4)	
Moderate		0 (0)	
Severe		0 (0)	

Values are mean ± SD or n (%).

LV = left ventricular; TAVR = transcatheter aortic valve replacement.

**Figure 3:**
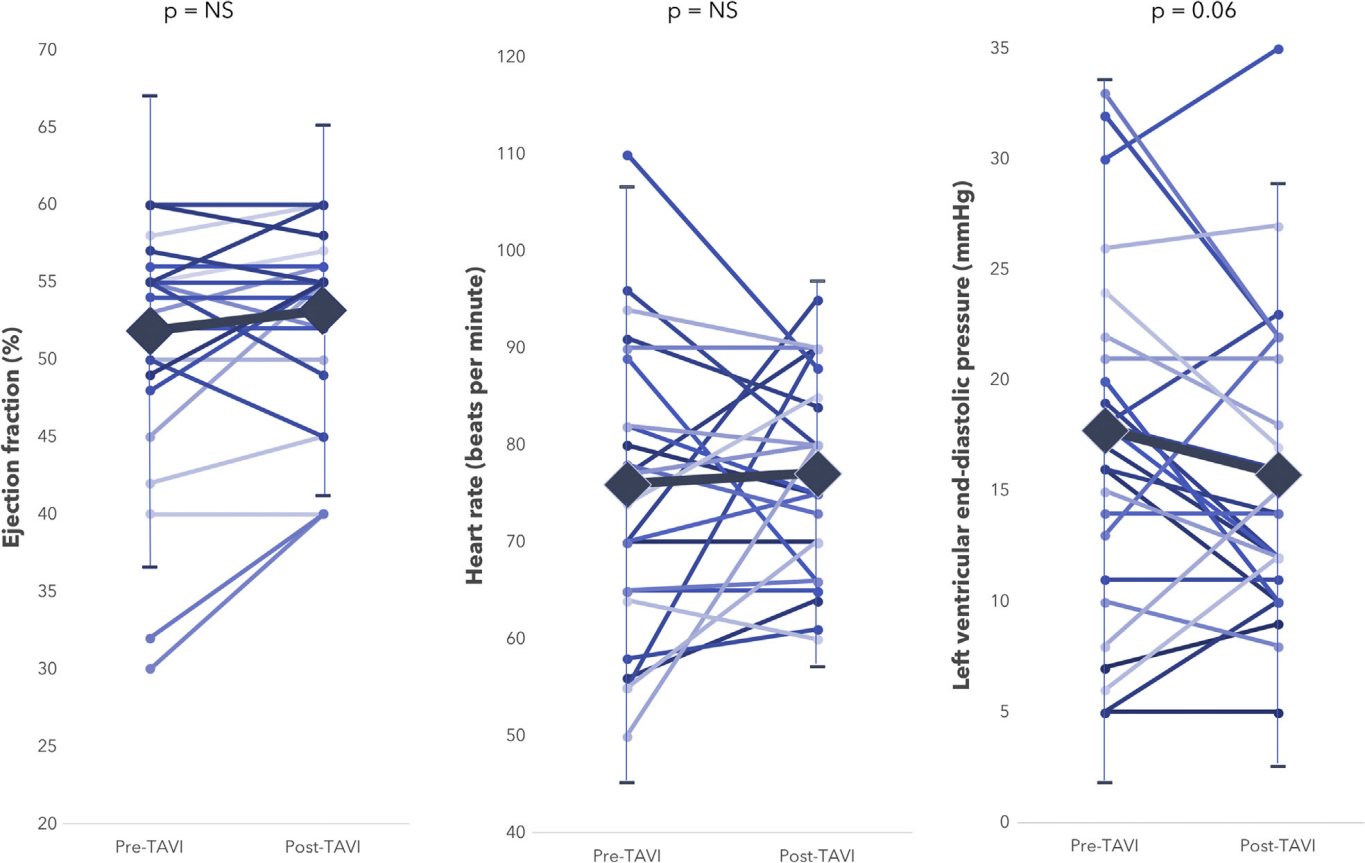
Figure Outlining the Changes in Ejection Fraction, Heart Rate, and Left Ventricular End-Diastolic Pressure Before and After Transcatheter Aortic Valve Replacement The **large diamonds** denote the mean values, with the **error bars** denoting the upper and lower 95% confidence intervals.

Pre-TAVR, LV systolic function was normal in 20 patients (71.4%), mildly impaired in 3 patients (10.7%), moderately impaired in 2 patients (7.1%), and severely impaired in 3 patients (10.7%). Post-TAVR, LV systolic function was normal in 20 patients (71.4%), mildly impaired in 3 patients (10.7%), moderately impaired in 4 patients (14.3%), and severely impaired in 1 patient (3.6%). Overall there was no significant difference in ejection fraction or heart rate post-TAVR, with a strong trend for reduction in LV end-diastolic pressure (p = 0.06) (Figure 3, Table 3).

Following TAVR, 15 patients (53.6%) had no paravalvular leak, 13 patients (46.4%) had trivial to mild paravalvular leak, and no patients had mild to moderate, moderate, or severe paravalvular leak (Table 3).

### Coronary flow pre- and post-TAVR

**Figure 4:**
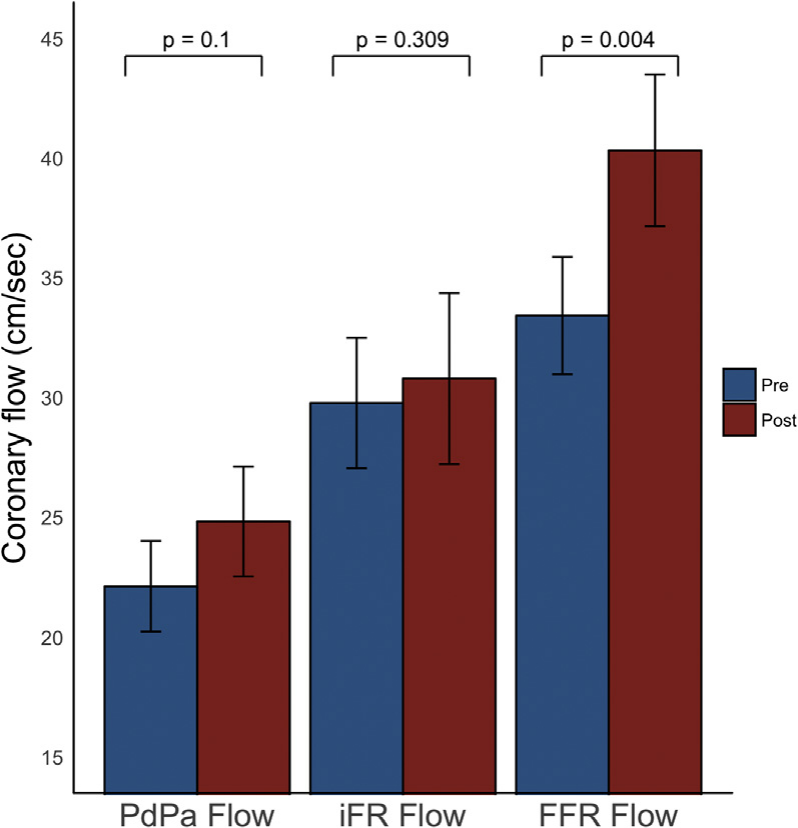
Coronary Flow Velocity Before and After Transcatheter Aortic Valve Replacement Figure demonstrating the changes in coronary flow before and after transcatheter aortic valve replacement (TAVR). The **left side of the graph** is resting flow over the whole cardiac cycle (PdPa-flow); the **middle side of the graph** is resting flow during the wave-period of diastole (iFR-flow); and the **right side of the graph** is hyperemic flow over the whole cardiac cycle (FFR-flow). Both PdPa-flow and FFR-flow increase significantly more post-TAVR than iFR-flow, which is constant. The **bars** denote mean values, with the **error bars** denoting SEs.

A summary of coronary hemodynamic parameters pre- and post-TAVR, under resting conditions and during hyperemia, is shown in Table 4. An example of invasive Doppler flow and pressure traces is shown in Figure 1. A summary of coronary flow pre- and post-TAVR is shown in Figure 4.

**Table 4:** Summary of Coronary Hemodynamic Variables at Rest and During Hyperemia Before and After Transcatheter Aortic Valve Replacement

	Resting			Hyperemia		
	Pre-TAVR	Post-TAVR	p Value	Pre-TAVR	Post-TAVR	p Value
Whole-cycle variables						
Flow velocity (cm/s)	22.13 ± 10.3	24.84 ± 12.5	0.10	33.44 ± 13.4	40.33 ± 17.4	0.004∗
Microvascular resistance (mm Hg · cm · s^−1^)	4.20 ± 1.9	4.14 ± 2.1	0.81	2.42 ± 0.9	2.14 ± 0.9	0.03∗
Aortic pressure (mm Hg)	85.85 ± 18.9	92.40 ± 1859	0.04∗	82.99 ± 18.0	88.44 ± 17.1	0.13
Systolic variables						
Flow velocity (cm/s)	16.48 ± 9.4	21.05 ± 13.1	0.004∗	27.67 ± 12.1	34.15 ± 17.5	0.01∗
Microvascular resistance (mm Hg · cm · s^−1^)	7.54 ± 3.8	6.60 ± 3.5	0.17	3.73 ± 1.6	3.45 ± 1.5	0.12
Aortic pressure (mm Hg)	101.46 ± 22.4	112.11 ± 24.	0.02∗	98.87 ± 22.7	110.55 ± 20.7	0.008∗
Wave-free variables						
Flow velocity (cm/s)	29.78 ± 14.9	30.81 ± 19.6	0.31	44.01 ± 20.6	42.52 ± 18.4	0.87
Microvascular resistance (mm Hg · cm · s^−1^)	2.59 ± 1.5	3.02 ± 1.6	0.02∗	1.53 ± 0.8	1.49 ± 0.6	0.52
Aortic pressure (mm Hg)	73.05 ± 15.1	76.41 ± 16.8	0.17	70.13 ± 16.3	70.69 ± 15.0	0.64
Diastolic variables						
Flow velocity (cm/s)	31.67 ± 15.4	33.33 ± 18.6	0.36	46.03 ± 20.5	45.94 ± 18.1	0.92
Microvascular resistance (mm Hg · cm · s^−1^)	2.65 ± 1.5	2.62 ± 1.3	0.92	1.50 ± 0.8	1.47 ± 0.6	0.63
Aortic pressure (mm Hg)	76.76 ± 16.6	78.13 ± 17.0	0.33	71.69 ± 14.9	74.02 ± 15.3	0.34

Values are mean ± SD.

TAVR = transcatheter aortic valve replacement.

∗ Statistically significant (p < 0.05).

#### Whole-cycle hemodynamic parameters

PdPa-flow increased nonsignificantly by 18.9 ± 4.4% post-TAVR (22.13 ± 10.3 cm/s pre-TAVR vs. 24.84 ± 12.5 cm/s post-TAVR; p = 0.10). FFR-flow increased by 25.0 ± 3.8% post-TAVR (33.44 ± 13.4 cm/s pre-TAVR vs. 40.33 ± 17.4 cm/s post-TAVR; p = 0.004).

#### Systolic hemodynamic parameters

Systolic resting flow increased by 36.8 ± 5.4% post-TAVR (16.48 ± 9.4 cm/s pre-TAVR vs. 21.05 ± 13.1 cm/s post-TAVR; p = 0.004). Systolic hyperemic flow increased by 31.2 ± 5.4% post-TAVR (27.67 ± 12.1 cm/s pre-TAVR vs. 34.15 ± 17.5 cm/s post-TAVR; p = 0.01).

#### Wave-free period hemodynamic parameters

There was no change in resting iFR-flow post-TAVR (29.78 ± 14.9 cm/s pre-TAVR vs. 30.81 ± 19.6 cm/s post-TAVR; p = 0.31). Hyperemic iFR-flow was also unchanged pre- and post-TAVR (44.01 ± 20.6 cm/s pre-TAVR vs. 42.52 ± 18.4 cm/s post-TAVR; p = 0.87).

### Comparison of PdPa-flow, iFR-flow, FFR-flow

Post-TAVR, PdPa-flow increased by 18.9 ± 4.4%, FFR-flow increased by 25.0 ± 3.8%, and iFR-flow increased by 5.7 ± 3.2%. PdPa-flow changed significantly more than iFR-flow (p = 0.01). FFR-flow also changed significantly more than iFR-flow (p < 0.0001) (Figure 4). The change in FFR-flow was similar to the change in PdPa-flow (p = 0.39).

### MVR pre- and post-TAVR

#### Whole-cycle hemodynamic parameters

Whole-cycle resting MVR was unchanged post-TAVR (4.20 ± 1.9 mm Hg · cm · s^−1^ pre-TAVR vs. 4.14 ± 2.1 mm Hg · cm · s^−1^ post-TAVR; p = 0.81). Whole-cycle hyperemic MVR decreased by 7.7% post-TAVR (2.42 ± 0.9 mm Hg · cm · s^−1^ pre-TAVR vs. 2.14 ± 0.9 mm Hg · cm · s^−1^ post-TAVR; p = 0.03).

#### Systolic hemodynamic parameters

Systolic resting MVR decreased numerically post-TAVR (7.54 ± 3.8 mm Hg · cm · s^−1^ pre-TAVR vs. 6.60 ± 3.5 mm Hg · cm · s^−1^ post-TAVR; p = 0.17). Systolic hyperemic MVR did not change post-TAVR (3.73 ± 1.6 mm Hg · cm · s^−1^ pre-TAVR vs. 3.45 ± 1.6 mm Hg · cm · s^−1^ post-TAVR; p = 0.12).

#### Wave-free period hemodynamic parameters

Wave-free resting MVR increased by 28.6% post-TAVR (2.59 ± 1.5 mm Hg · cm · s^−1^ pre-TAVR vs. 3.02 ± 1.6 mm Hg · cm · s^−1^ post-TAVR; p = 0.02). Wave-free hyperemic MVR was constant post-TAVR (1.53 ± 0.8 mm Hg · cm · s^−1^ pre-TAVR vs. 1.49 ± 0.6 mm Hg · cm · s^−1^ post-TAVR; p = 0.52).

### Indices of coronary stenosis severity before and after TAVR

**Figure 5:**
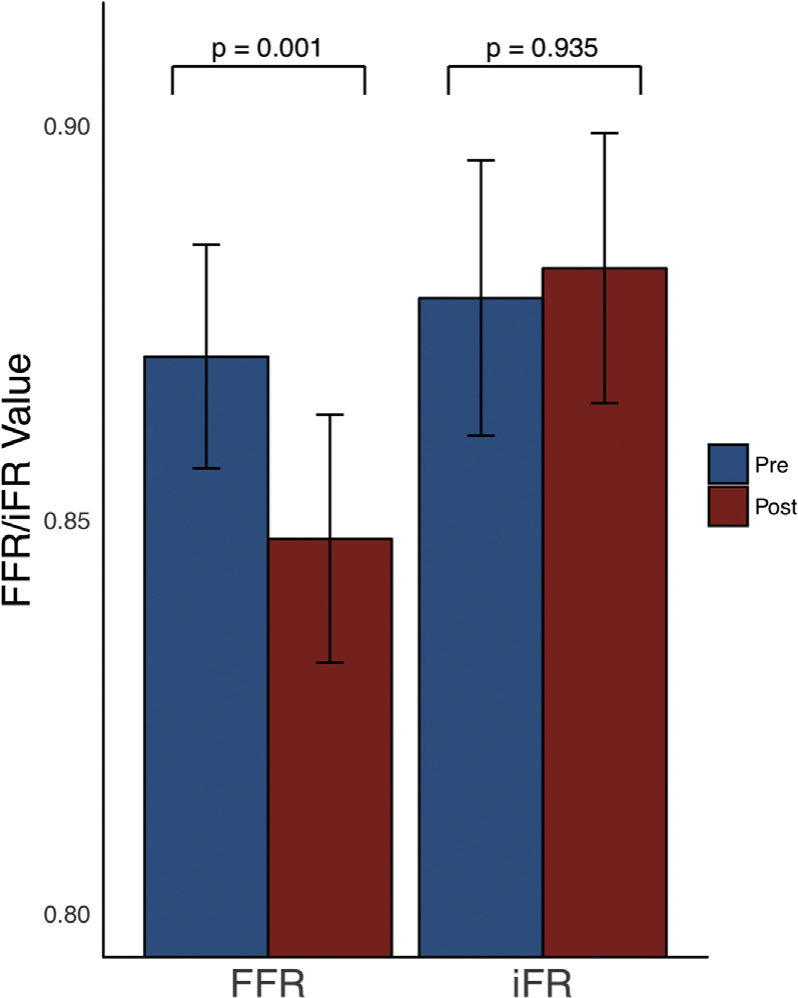
Changes in Fractional Flow Reserve and Instantaneous Wave-Free Ratio After Transcatheter Aortic Valve Replacement Figure demonstrating the change in fractional flow reserve (FFR) and instantaneous wave-free ratio (iFR) values after transcatheter aortic valve replacement. FFR decreases significantly, whereas iFR remains constant. The **bars** denote mean values, with the **error bars** denoting SEs.

iFR values did not change post TAVR (0.88 ± 0.09 pre-TAVR vs. 0.88 ± 0.09 post-TAVR; p = 0.94) (Figure 5). FFR values significantly decreased after TAVR (0.87 ± 0.08 pre-TAVR vs. 0.85 ± 0.09 post-TAVR; p = 0.0008). Basal stenosis resistance values did not change post-TAVR (0.31 ± 0.29 pre-TAVR vs. 0.32 ± 0.26 post-TAVR; p = 0.5). Hyperemic stenosis resistance values increased after TAVR (0.34 ± 0.32 pre-TAVR vs. 0.40 ± 0.32 post-TAVR; p = 0.06). A summary of the indices of coronary stenosis severity before and after TAVR is shown in Table 5.

**Table 5:** Indices of Coronary Stenosis Severity Before and After Transcatheter Aortic Valve Replacement

	Pre-TAVR	Post-TAVR	p Value
Hyperemic indices			
Fractional flow reserve	0.87 ± 0.08	0.85 ± 0.09	0.0008
Hyperemic stenosis resistance	0.34 ± 0.32	0.40 ± 0.32	0.06
Resting indices			
Instantaneous wave-free ratio	0.88 ± 0.09	0.88 ± 0.09	0.94
Basal stenosis resistance	0.31 ± 0.29	0.32 ± 0.26	0.50
Pd/Pa	0.91 ± 0.29	0.92 ± 0.06	0.82
Diastolic Pd/Pa	0.88 ± 0.10	0.89 ± 0.09	0.75

Values are mean ± SD.

TAVR = transcatheter aortic valve replacement.

## Discussion

In this study, we have shown that 1) iFR-flow does not change post-TAVR; 2) FFR-flow increases significantly post-TAVR; 3) changes in FFR-flow are driven by significant increases in systolic flow post-TAVR; and 4) iFR values do no change post-TAVR, whereas FFR decreases significantly post-TAVR.

### Phasic coronary flow in patients with severe AS

Coronary flow is phasic and occurs in both systole and diastole. Systolic flow is driven predominantly by pressure changes at the aortic end of the vessel (13). Diastolic flow is driven by pressure changes at the distal end of the vessel, due to contraction and relaxation of the myocardium and its interaction with the microcirculation 14, 15, 16.

**Figure 6:**
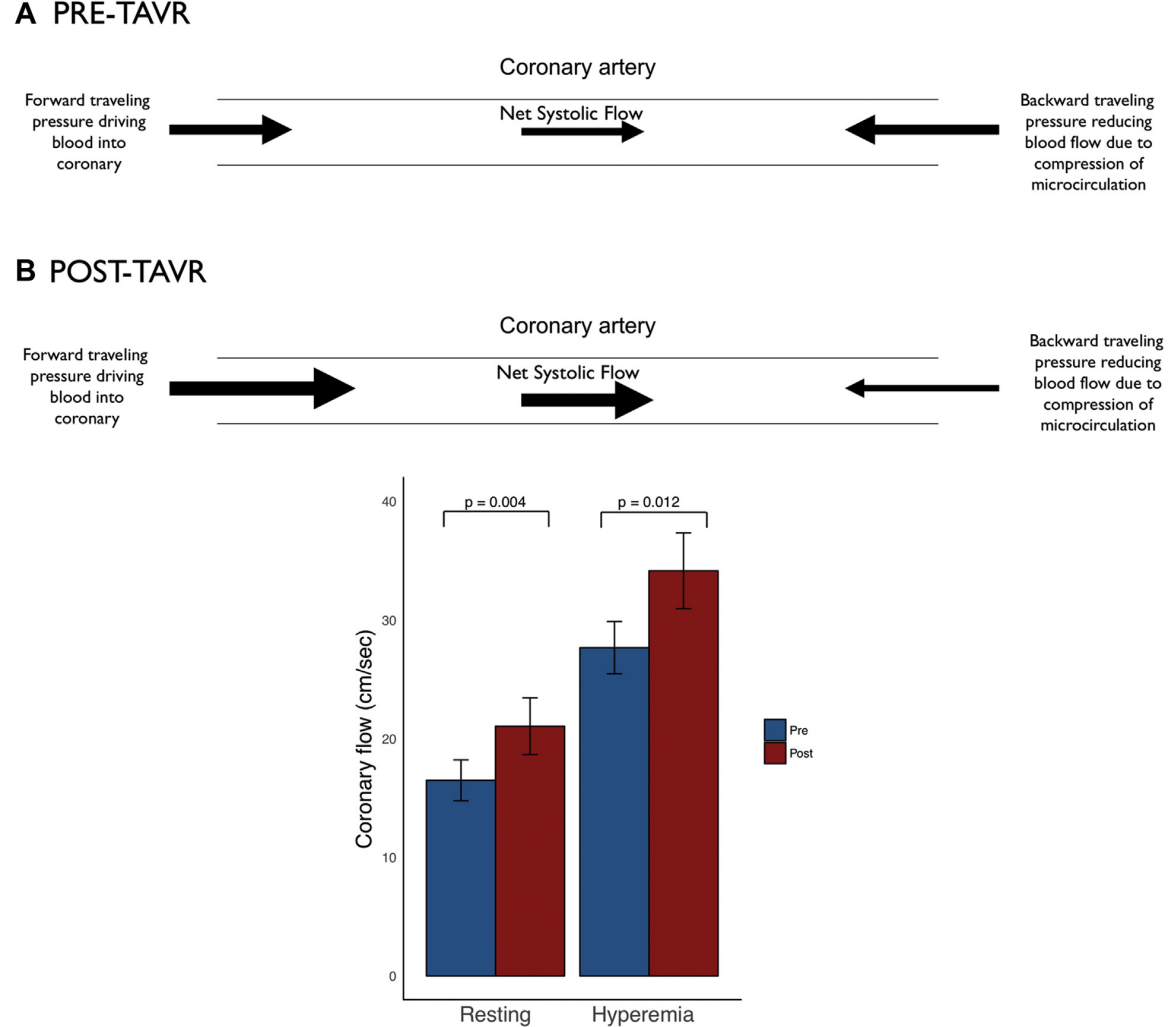
Changes in Systolic Coronary Flow After Transcatheter Aortic Valve Replacement Figure demonstrating the changes in systolic coronary flow after transcatheter aortic valve replacement (TAVR). **(A)** There is a schematic, demonstrating that post-TAVR there is increase in the forward traveling systolic pressure, leading to an increase in systolic coronary flow. There is also a reduction in the compressive forces on the microcirculation post-TAVR; these 2 factors both contribute to a net increase in systolic coronary flow post-TAVR. **(B)** Statistically significantly increase in systolic coronary flow seen in our study, both at rest and during Hyperemia. The **bars** denote mean values, with the **error bars** denoting SEs.

During systole, coronary flow is a function of blood emptying from the left ventricle through the aortic valve into the aorta and the opposing compression forces from the contracting myocardium, which blunts systolic flow. In severe AS, systolic coronary flow is reduced because of obstruction of ventricular emptying by the stenosed aortic valve and simultaneous compression of the microcirculation from the contracting myocardium opposing forward flow in the coronary artery, which is augmented by the elevated intraventricular pressure in patients with severe AS 17, 18. This results in a reduction in coronary flow during systole. Treatment of the valve stenosis by TAVR removes the mechanical obstruction to ventricular emptying, increases aortic flow, and reduces intraventricular pressure and therefore increases systolic coronary flow (19). This significant systolic increment in flow post-TAVR occurs at rest and during hyperemia (Figure 6).

Diastolic flow during the wave-free period occurs when the myocardium is neither contracting nor actively relaxing (12). During this period, the aortic valve is closed. Restriction of aortic valve opening, a systolic phenomenon, therefore does not affect flow during the wave-free period of diastole, because regardless of the severity of AS, the aortic valve leaflets are closed and therefore the aortic valve is not actively contributing to coronary flow.

### The effect of aortic stenosis on hyperemic flow

Maximal blood flow in a coronary artery is affected by microvascular structure, function, LV end-diastolic pressure, and right atrial pressure 20, 21, 22, 23. Any condition that affects 1 of these determinants will affect maximal flow. In AS, LV afterload is increased because of the stenosed valve (24). This results in raised LV end-diastolic pressure and LV hypertrophy, leading to structural changes in the microcirculation that affect its ability to respond to hyperemic agents (25). Furthermore, patients with severe AS have increased circulating vasoconstrictors as part of a compensatory mechanism to increase vascular tone and maintain systemic blood pressure (26). These vasoconstrictors counteract the effect of administered vasodilators such as adenosine and may therefore also attenuate the response of the coronary microcirculation to adenosine.

The protocol in this study permitted the isolation of the acute effect of treating a stenosed aortic valve on coronary hemodynamics. This study demonstrates that hyperemic flow increases significantly post-TAVR. This is driven by a significant increase in the systolic component of flow. In contrast, flow during the wave-free period does not change during hyperemia post TAVR, which is consistent with the minimal effect of the aortic valve on coronary flow during this period. Therefore, any index of coronary flow that includes the systolic phase of the cardiac cycle will be susceptible to change post-TAVR. In contrast, indices of flow that do not involve systole may be less vulnerable to restriction of aortic valve opening.

### Indices of coronary stenosis severity and aortic stenosis

A significant proportion of patients with severe AS also have concomitant CAD (2). The assessment of this disease is challenging, and the established hyperemia-based indices of coronary stenosis severity have not been validated in this setting. Extrapolation of FAME (Fractional Flow Reserve Versus Angiography for Multivessel Evaluation) (4) data would suggest that treatment based on coronary angiography alone is likely to lead to unnecessary revascularization. In addition, coronary intervention is not without risk in patients with severe AS.

The 2 most clinically applicable and validated indices of coronary stenosis severity are FFR and iFR. Although these are both pressure-derived indices of stenosis severity, their physiological principles rely on the fact that pressure is proportional to underlying coronary flow during their measurement. Therefore, any change in coronary flow will lead to a change in the pressure-only index.

**Figure 7:**
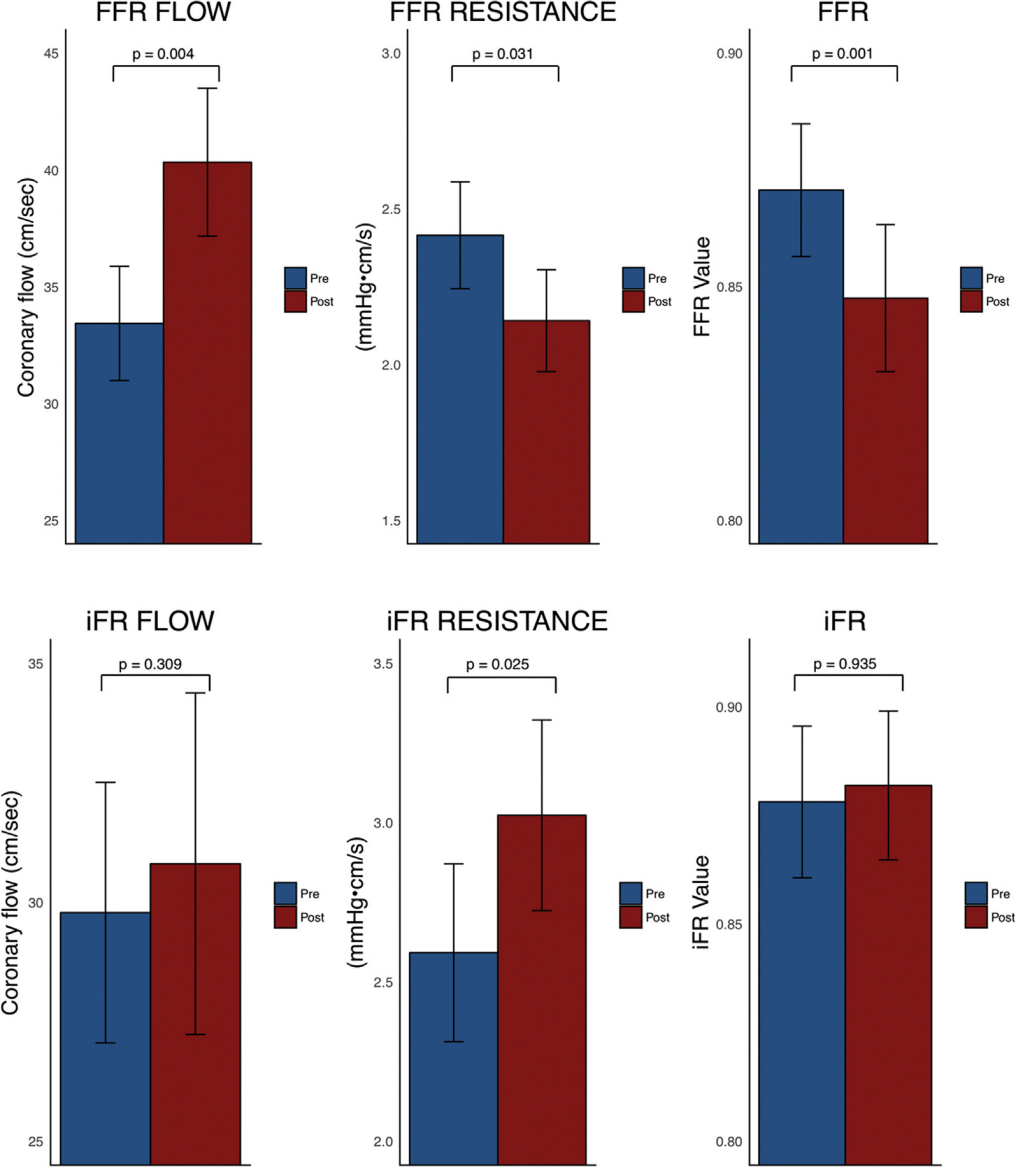
Coronary Hemodynamic Status Before and After Transcatheter Aortic Valve Replacement, Over Both the Fractional Flow Reserve and Instantaneous Wave-Free Ratio Measurement Windows Figure demonstrating the changes in coronary hemodynamic status over the fractional flow reserve (FFR) and instantaneous wave-free ratio (iFR) measurement windows. The **top row** shows the changes in coronary hemodynamics over the FFR window (the whole cardiac cycle during hyperemia): the **left panel** demonstrates a significant increase in flow, the **middle panel** demonstrates a significant reduction in resistance; and as a consequence the **right panel** demonstrates a significant reduction in the FFR value after transcatheter aortic valve replacement (TAVR). The **bottom row** shows the changes in coronary hemodynamic status over the iFR window (the wave-free period of diastole at rest): the **left panel** demonstrates constant flow before and after TAVR; the **right panel** demonstrates a constant iFR value post-TAVR; to achieve the same pressure gradient with the same flow velocity, there is therefore a significant increase in resistance (shown in the **middle panel**). The **bars** denote mean values, with the **error bars** denoting SEs.

FFR is measured over the whole cardiac cycle. As a result, it includes systolic flow. The significant change in hyperemic systolic flow immediately after TAVR has a significant effect on whole-cycle flow and therefore FFR. The blunted whole-cycle hyperemic flow pre-TAVR leads to FFR’s systematically underestimating coronary stenosis severity in the presence of AS, with an increase in hyperemic flow post-TAVR resulting in FFR values becoming significantly lower across the same coronary stenosis (Figure 7).

iFR is a nonhyperemic index of stenosis severity that is measured during the diastolic wave-free period (6). During this period pressure and flow are proportional. We demonstrate that this diastolic wave-free period exists in patients with severe AS. Furthermore, coronary flow during the diastolic wave-free period does not change post-TAVR, indicating its relative independence from the acute relief of AS. This ability to discriminate the coronary stenosis severity from AS appears to be true of this period at rest and during hyperemia. The consistency of flow during this period post-TAVR means that, in contrast to FFR, the iFR value does not change post-TAVR (Figure 7).

These phenomena can also be observed by comparing the results of our study with those of other studies on indices of coronary stenosis severity in patients without severe AS. In the DEFINE-FLAIR (Functional Lesion Assessment of Intermediate Stenosis to Guide Revascularisation) and iFR-SWEDEHEART (Evaluation of iFR vs FFR in Stable Angina or Acute Coronary Syndrome) trials, the mean iFR values were 0.91 ± 0.09 and 0.91 ± 0.10, respectively, similar to the mean iFR of 0.88 ± 0.09 seen in this study. The mean FFR values, however, were 0.83 ± 0.09 in DEFINE-FLAIR and 0.82 ± 0.10 in iFR-SWEDEHEART, lower than those seen in this study (0.87 ± 0.08). This is a function of the attenuated hyperemia in these patients, due to a blunted effect of adenosine resulting in failure to augment flow sufficiently to produce FFR values similar to those in patients without severe AS.

There is a paucity of available data regarding coronary stenosis assessment in patients with severe AS. Existing studies have not measured coronary flow and assumed that it is not affected by AS 27, 28, 29. The present study demonstrates that the effect of adenosine is significantly altered in the presence of AS, and this will consequently significantly affect FFR values and therefore any FFR treatment threshold. The significantly blunted effect of adenosine in these patients suggests that the fundamental intracoronary conditions for accurate FFR assessment cannot be met in patients with severe AS and therefore calls into question the role of FFR as an ischemic standard in these patients (30).

### Clinical implications

The findings of this study have potential implications for patients with severe AS and coronary disease who are undergoing TAVR. The ability to isolate coronary stenosis severity in the context of AS will allow clinicians to determine in which patients the valve alone can be treated and which patients need concomitant revascularization, which may be via angioplasty or, in conventional surgical aortic valve replacement, bypass surgery. Hyperemic indices that include systole, such as FFR, are unable to accurately determine coronary stenosis severity in this setting, because of a blunted hyperemic response, suggesting that potentially flow-limiting coronary lesions may be denied appropriate treatment. The degree of AS at which hyperemic flow begins to reduce is also unknown, raising the possibility maximal hyperemia is not achievable in patients with moderate or even mild AS. Furthermore, the variable and unpredictable rate of regression of LV hypertrophy also suggests that FFR may still be vulnerable to an inability to achieve maximal hyperemia for several months after valve treatment.

Flow during the wave-free period of diastole is independent of the severity of the AS, suggesting that iFR can be used to accurately discriminate coronary stenosis severity in the setting of AS. Further studies are required to determine if there is any significant effect of LV hypertrophy regression on iFR values in this setting. The true of role of iFR in patients will be appreciated only with a prospective study comparing an iFR-guided approach to revascularization to standard angiographically guided therapy in patients with severe AS.

### Study limitations

This study included patients with severe symptomatic AS for whom TAVR was decreed the most appropriate therapy by the heart team, in accordance with international guidelines (3). Our results cannot therefore be generalized to patients with more mild degrees of AS.

Adenosine was administered as an intracoronary bolus and not via intravenous infusion. We cannot therefore exclude the possibility that intravenous adenosine infusion would yield different results. However, intracoronary adenosine is recognized as a valid approach to FFR assessment 31, 32, and such assessments have been included in all the large randomized trials of physiology to date 33, 34. Intravenous infusion was avoided because of the recognized potential for a 15% reduction in aortic pressure (35) that could potentially destabilize a patient with severe AS.

Post-TAVR physiological measurements were made immediately after the valve had been replaced and within the same catheter laboratory procedure. We cannot therefore comment on any more long-term changes in coronary hemodynamics.

The prevalence of severe aortic regurgitation has been significantly reduced with the development of the current generation of TAVR valves (36). This is reflected in the presence of only trivial to mild aortic regurgitation in our dataset compared with other groups that used earlier generation valves 37, 38. It is therefore unlikely that the degree of AR, which was mild at most in a minority of our patients, would explain the large differences seen in this study between systolic and diastolic parameters and hyperemia and resting parameters.

The sample size of our study may be considered small, with 30 coronary lesions across 28 patients. However, this is the largest study to date of invasive coronary flow in patients with severe AS and the first to study patients with stenosed coronary arteries. It is also the first study to include phasic analysis, permitting an increase in our understanding of the coronary physiology in this complex hemodynamic condition. This was a mechanistic study, aiming to provide a comprehensive insight to coronary hemodynamic status in patients with severe AS undergoing TAVR. A decision-making strategy for revascularization in patients with severe AS, on the basis of current FFR or iFR data, cannot be made.

This study was designed to compare hyperemic and resting coronary flow and to perform a phasic analysis to delineate differences between systole and diastole. It was not, however, powered to detect differences between resting indices of coronary stenosis severity. Our phasic analysis suggests that there is a significant change in systolic flow post-TAVR, during both resting conditions and hyperemia. This suggests that if the sample size were increased, we may see significant differences between whole cycle and diastolic resting indices.

## Conclusions

Systolic coronary flow and hyperemic coronary flow are significantly reduced in severe AS and change significantly post-TAVR. Hyperemic indices that include systole therefore provide a limited assessment of true coronary stenosis severity in patients with severe AS. Flow during the wave-free period of diastole does not change post TAVR, suggesting that, in patients with severe AS, coronary indices calculated during this period may be more reflective of true coronary stenosis severity.

Perspectives **WHAT IS KNOWN?** A significant proportion of patients with severe AS have concomitant CAD. There is no established index of coronary stenosis severity in these patients.

**WHAT IS NEW?** Systolic coronary flow and hyperemic coronary flow are significantly reduced in severe AS and change significantly post-TAVR, making indices of coronary stenosis severity that include systole and are made during hyperemic conditions unreliable in this context. Coronary flow during the wave-free period of diastole does not change post-TAVR, therefore indices restricted to this period are more accurate in patients with severe AS.

**WHAT IS NEXT?** Prospective randomized trials of coronary revascularization in patients with severe AS are required to determine the optimal method of assessing and treating CAD in this cohort.

## References

[1] Stewart B.F., Siscovick D., Lind B.K. (1997). Clinical factors associated with calcific aortic valve disease. Cardiovascular Health Study. J Am. Coll Cardiol.

[2] Iung B. (2000). Interface between valve disease and ischaemic heart disease. Heart Br Card Soc.

[3] Vahanian A., Joint Task Force on the Management of Valvular Heart Disease of the European Society of Cardiology (ESC), European Association for Cardio-Thoracic Surgery (EACTS) (2012). Guidelines on the management of valvular heart disease (version 2012). Eur Heart J.

[4] Tonino P.A.L., De Bruyne B., Pijls N.H.J. (2009). Fractional flow reserve versus angiography for guiding percutaneous coronary intervention. N Engl J Med.

[5] Pijls N.H., De Bruyne B., Peels K. (1996). Measurement of fractional flow reserve to assess the functional severity of coronary-artery stenoses. N Engl J Med.

[6] Sen S., Escaned J., Malik I.S. (2012). Development and validation of a new adenosine-independent index of stenosis severity from coronary wave-intensity analysis: results of the ADVISE (Adenosine Vasodilator Independent Stenosis Evaluation) study. J Am Coll Cardiol.

[7] Davies J.E., Sen S., Broyd C. (2011). Arterial pulse wave dynamics after percutaneous aortic valve replacement: fall in coronary diastolic suction with increasing heart rate as a basis for angina symptoms in aortic stenosis. Circulation.

[8] Verhoeff B.-J., Siebes M., Meuwissen M. (2005). Influence of percutaneous coronary intervention on coronary microvascular resistance index. Circulation.

[9] Sen S., Asrress K.N., Nijjer S. (2013). Diagnostic classification of the instantaneous wave-free ratio is equivalent to fractional flow reserve and is not improved with adenosine administration. Results of CLARIFY (Classification Accuracy of Pressure-Only Ratios Against Indices Using Flow Study). J Am Coll Cardiol.

[10] van de Hoef T.P., Nolte F., Damman P. (2012). Diagnostic accuracy of combined intracoronary pressure and flow velocity information during baseline conditions: adenosine-free assessment of functional coronary lesion severity. Circ Cardiovasc Interv.

[11] Meuwissen M., Siebes M., Chamuleau S.A.J. (2002). Hyperemic stenosis resistance index for evaluation of functional coronary lesion severity. Circulation.

[12] Davies J.E., Whinnett Z.I., Francis D.P. (2006). Evidence of a dominant backward-propagating “suction” wave responsible for diastolic coronary filling in humans, attenuated in left ventricular hypertrophy. Circulation.

[13] Krams R., Sipkema P., Zegers J., Westerhof N. (1989). Contractility is the main determinant of coronary systolic flow impediment. Am J Physiol.

[14] Spaan J.A., Breuls N.P., Laird J.D. (1981). Diastolic-systolic coronary flow differences are caused by intramyocardial pump action in the anesthetized dog. Circ Res.

[15] Wiggers C.J. (1954). The interplay of coronary vascular resistance and myocardial compression in regulating coronary flow. Circ Res.

[16] Krams R., Sipkema P., Westerhof N. (1989). Varying elastance concept may explain coronary systolic flow impediment. Am J Physiol.

[17] Hongo M., Goto T., Watanabe N. (1993). Relation of phasic coronary flow velocity profile to clinical and hemodynamic characteristics of patients with aortic valve disease. Circulation.

[18] Spaan J.A. (1995). Mechanical determinants of myocardial perfusion. Basic Res Cardiol.

[19] Fujiwara T., Nogami A., Masaki H. (1989). Coronary flow velocity waveforms in aortic stenosis and the effects of valve replacement. Ann Thorac Surg.

[20] Layland J., Wilson A.M., Whitbourn R.J. (2013). Impact of right atrial pressure on decision-making using fractional flow reserve (FFR) in elective percutaneous intervention. Int J Cardiol.

[21] Spaan J.A.E., Piek J.J., Hoffman J.I.E., Siebes M. (2006). Physiological basis of clinically used coronary hemodynamic indices. Circulation.

[22] van de Hoef T.P., Meuwissen M., Escaned J. (2013). Fractional flow reserve as a surrogate for inducible myocardial ischaemia. Nat Rev Cardiol.

[23] van de Hoef T.P., Nolte F., Rolandi M.C. (2012). Coronary pressure-flow relations as basis for the understanding of coronary physiology. J Mol Cell Cardiol.

[24] Broyd C.J., Sen S., Mikhail G.W., Francis D.P., Mayet J., Davies J.E. (2013). Myocardial ischemia in aortic stenosis: insights from arterial pulse-wave dynamics after percutaneous aortic valve replacement. Trends Cardiovasc Med.

[25] Dixon J.A., Spinale F.G. (2011). Myocardial remodeling: cellular and extracellular events and targets. Annu Rev Physiol.

[26] Heusch G. (2010). Alpha-adrenergic coronary vasoconstriction in humans. J Am Coll Cardiol.

[27] Pesarini G., Scarsini R., Zivelonghi C. (2016). Functional assessment of coronary artery disease in patients undergoing transcatheter aortic valve implantation: influence of pressure overload on the evaluation of lesions severity. Circ Cardiovasc Interv.

[28] Scarsini R., Pesarini G., Zivelonghi C. (2017). Coronary physiology in patients with severe aortic stenosis: comparison between fractional flow reserve and instantaneous wave-free ratio. Int J Cardiol.

[29] Scarsini R., Pesarini G., Zivelonghi C. (2018). Physiologic evaluation of coronary lesions using instantaneous wave-free ratio (iFR) in patients with severe aortic stenosis undergoing transcatheter aortic valve implantation. EuroIntervention.

[30] Sen S., Ahmad Y., Davies J. (2018). Assessing coronary disease in patients with severe aortic stenosis: the need for a “valid” gold standard for validation studies?. EuroIntervention.

[31] De Bruyne B., Gould K.L. (2013). Standardized hyperemic stress for fractional flow reserve. Circ Cardiovasc Interv.

[32] Li J., Elrashidi M.Y., Flammer A.J. (2013). Long-term outcomes of fractional flow reserve-guided vs. angiography-guided percutaneous coronary intervention in contemporary practice. Eur Heart J.

[33] Davies J.E., Sen S., Dehbi H.-M. (2017). Use of the instantaneous wave-free ratio or fractional flow reserve in PCI. N Engl J Med.

[34] Götberg M., Christiansen E.H., Gudmundsdottir I.J. (2017). Instantaneous wave-free ratio versus fractional flow reserve to guide PCI. N Engl J Med.

[35] Tarkin J.M., Nijjer S., Sen S. (2013). Hemodynamic response to intravenous adenosine and its effect on fractional flow reserve assessment: results of the Adenosine for the Functional Evaluation of Coronary Stenosis Severity (AFFECTS) study. Circ Cardiovasc Interv.

[36] Wiegerinck E.M.A., van de Hoef T.P., Rolandi M.C. (2015). Impact of aortic valve stenosis on coronary hemodynamics and the instantaneous effect of transcatheter aortic valve implantation. Circ Cardiovasc Interv.

[37] Leon M.B., Smith C.R., Mack M. (2010). Transcatheter aortic-valve implantation for aortic stenosis in patients who cannot undergo surgery. N Engl J Med.

[38] Reardon M.J., Van Mieghem N.M., Popma J.J. (2017). Surgical or transcatheter aortic-valve replacement in intermediate-risk patients. N Engl J Med.

